# Concomitant use of dextromethorphan and alcohol‐induced dissociation in a patient with alcohol dependence

**DOI:** 10.1002/pcn5.70011

**Published:** 2024-10-04

**Authors:** Yasuhito Nagai, Yuko Tajima, Koichi Miyakawa

**Affiliations:** ^1^ Department of Psychiatry Juntendo University Graduate School of Medicine Tokyo Japan; ^2^ Department of Psychiatry Juntendo University Urayasu Hospital Urayasu Japan

**Keywords:** alcohol dependence, dextromethorphan, over‐the‐counter (OTC) drug

## Abstract

**Background:**

Dextromethorphan (DXM) is an over‐the‐counter drug that is commonly abused by teenagers. Its main pharmacological property is a low‐affinity *N*‐methyl‐d‐aspartate (NMDA) receptor antagonist.

**Case Presentation:**

We report acute psychotic dissociation induced by DXM and alcohol in a patient with alcohol dependence, which made it difficult to distinguish alcohol withdrawal symptoms with limited information. The psychotic symptoms were ameliorated by haloperidol; however, in cases of DXM abuse, haloperidol treatment should be avoided.

**Conclusion:**

In the consultation of patients with addiction, the possibility of various addictive drugs must be kept in mind. The concomitant use of DXM and alcohol causes easy dissociation and high‐dose usage of DXM and alcohol withdrawal symptoms are difficult to distinguish.

## BACKGROUND

Abuse of over‐the‐counter (OTC) drugs is a well‐recognized social problem in adolescence. Since 2018 in Japan, the most abused drugs among teenagers are OTC drugs.^1^ Dextromethorphan (DXM) is an OTC drug whose annual rate of intentional abuse has tripled from 2000–2006 to 2006–2015 in the United States.^2^ We treated a patient with alcohol dependence who experienced acute psychotic dissociation induced by DXM and alcohol, which made it difficult to distinguish alcohol withdrawal symptoms (AWS) under the limited information. We report this case presentation to raise awareness about the hazards of OTC drugs and to discuss clinical pharmacological pathology. The patient provided written informed consent for the publication of this case.

## CASE PRESENTATION

The case was a 27‐year‐old male with alcohol dependence, hyperlipidemia, and hyperuricemia. At the age of 20, he began habitual drinking. At the age of 22, because of the COVID‐19 pandemic, he was fired, and the quantity of alcoholic drinks was increased to 128 g of pure alcohol per day. At the age of 23, he worked as a host in a nightclub, and he drank heavily and sometimes took cannabis. After 6 months, he visited a hospital to present tremors in interval periods, and insomnia. Because he was diagnosed with alcohol dependence according to the *International Statistical Classification of Diseases and Related Health Problems*, tenth revision,^3^ he was admitted to a hospital to attend an alcohol rehabilitation program. However, he could not bear the environment and group living, and was discharged from the hospital without completing the program. After discharge, he continued outpatient visits to be prescribed zolpidem 5 mg for insomnia. At the age of 26, he visited our hospital for the first time. He was unemployed and drank every day. Blood samples showed liver and kidney function failure. We started acamprosate 999 mg/day medication and motivational therapy. After medication, the drink amount decreased up to 64 g of pure alcohol per day. However, the patient thought that it was better to take dissociative drugs than to drink alcohol and took a lot of OTC drugs. He was addicted to DXM and SS‐bron, which contains dihydrocodeine, dl‐ethylephedrine, and anhydrous caffeine; however, this abuse was kept a secret, and we did not know until his confession after the following episode. At the age of 27, he took DXM 600 mg to feel euphoria and dissociation on April 25 and 29. This time, he felt euphoria and visual hallucinations without hyperthermia and diaphoresis. On May 8, he drank 32 g of pure alcohol in the morning, and 2 h later, he took DXM 300 mg. Because of psychotic features, including visual hallucination and excitement, his parents called an ambulance. He arrived at our hospital for an emergency visit. The body temperature was 38.3°C, and the systolic blood pressure was 160 mmHg. He presented dissociation, ataxia, and diaphoresis. Because of his screaming and excitement, haloperidol 5 mg by intravenous administration was used. After 1 h, his hallucination and excitement were ameliorated. Because we did not know about his DXM abuse at this time, we thought that the symptoms were due to alcohol withdrawal delirium and administered diazepam 10 mg by intramuscular injection. After the transfusion, all his symptoms were ameliorated, and he returned home. On the next visit, he confessed to DXM abuse. We could diagnose acute psychosis or dissociation by the drug. After careful discussion during outpatient visits, the patient now attends an addiction therapy hospital for educational treatment and quit the addictive agents during hospitalization.

## DISCUSSION

DXM has multiple pharmacological properties. It primarily acts as a low‐affinity *N*‐methyl‐d‐aspartate (NMDA) receptor antagonist, leading to euphoria, agitation, hallucination, dissociation, and coma, and as a weaker agonist of mu‐opioid receptors. The antitussive effect of DXM is a binding sigma opioid receptor in the medulla. In addition, dextrorphan, which is one of the metabolites of DXM, acts as an inhibiting serotonin transporter and noradrenaline reuptake, and blocks alpha3‐beta‐4 nicotinic receptors. Besides, dextrorphan is a stronger NMDA receptor antagonist and a weaker sigma‐1 receptor agonist than DXM. Therefore, a high dose of DXM causes not only psychosis/dissociation but also hypertension, ataxia, and serotonin syndrome.^4^ DXM dosages between 100 mg and 400 mg can induce euphoria, while those of 600–1500 mg can induce dissociation and hallucination,^5^ suggesting that alcohol use enhances the adverse effect of DXM. Indeed, concomitant use of DXM and other drugs, including alcohol, was common in deceased abusers of DXM^6^ and 10% of opioid dependents on maintenance therapy reported DXM abuse.^7^ In this case, the patient denied current cannabis use, but he had a history of cannabis use. Alcohol acts as an agonist γ‐aminobutyric acid (GABA) receptor. Chronic alcohol consumption leads to a down‐regulation of GABAergic receptors and up‐regulation of NMDA receptors in patients with alcohol dependence, indicating that acute discontinuation of alcohol may cause reduced GABAergic inhibition and NMDA overexcitation contributing to AWS.^8^ In point of NMDA excitation, high‐dose usage of DXM and AWS are difficult to distinguish without medication history information. DXM shares pharmacological properties with acamprosate and nalmefene; however, lack of efficacy in AWS was confirmed.^9^ In the literature, DXM‐induced psychosis was treated by antipsychotics, including haloperidol, risperidone, quetiapine, and olanzapine.^10^, ^11^ DXM metabolism is mediated through the isozyme group CYP2D6. Although we used haloperidol for the treatment of acute psychosis, haloperidol metabolites inhibit the CYP2D6 enzyme and there is a possibility that haloperidol treatment increases DXM blood concentration and exacerbates hyperthermia and anticholinergic symptoms.^4^, ^12^ If prior information of DXM abuse is available or in the case of DXM overdose, haloperidol should be avoided. Considering the common abuse of DXM and that most DXM dependents do not see doctors, complete remission without neuroleptics after discontinuing the abuse of DXM is possible. If antipsychotics are used for the treatment of DXM intoxication, low doses of risperidone and olanzapine seem to be tolerated by the previous case reports.^4^, ^11^, ^13^

## CONCLUSION

In the consultation of patients with addiction, the possibility of various addictive drugs must be kept in mind. The concomitant use of DXM and alcohol causes easy dissociation and high‐dose usage of DXM and AWS are difficult to distinguish.

## AUTHOR CONTRIBUTIONS

Yasuhito Nagai and Yuko Tajima treated the patient. Yasuhito Nagai wrote the original manuscript. All authors participated in discussion, writing this manuscript, and revisions. All read and approved the final version of the manuscript.

## CONFLICT OF INTEREST STATEMENT

The authors declare no conflict of interest.

## ETHICS APPROVAL STATEMENT

N/A

## PATIENT CONSENT STATEMENT

The patient provided written informed consent for the publication of this case.

## CLINICAL TRIAL REGISTRATION

N/A

## Data Availability

N/A
